# Metabolomics analysis reveals Embden Meyerhof Parnas pathway activation and flavonoids accumulation during dormancy transition in tree peony

**DOI:** 10.1186/s12870-020-02692-x

**Published:** 2020-10-23

**Authors:** Tao Zhang, Yanchao Yuan, Yu Zhan, Xinzhe Cao, Chunying Liu, Yuxi Zhang, Shupeng Gai

**Affiliations:** 1 College of Life Sciences, Qingdao Agricultural University, Qingdao, 266109 China; 2 University Key Laboratory of Plant Biotechnology in Shandong Province, Qingdao, 266109 China

**Keywords:** Tree peony, Dormancy transition, Metabolomics, EMP activation, Flavonoids accumulation

## Abstract

**Background:**

Bud dormancy is a sophisticated strategy which plants evolve to survive in tough environments. Endodormancy is a key obstacle for anti-season culture of tree peony, and sufficient chilling exposure is an effective method to promote dormancy release in perennial plants including tree peony. However, the mechanism of dormancy release is still poorly understood, and there are few systematic studies on the metabolomics during chilling induced dormancy transition.

**Results:**

The tree peony buds were treated with artificial chilling, and the metabolmics analysis was employed at five time points after 0–4 °C treatment for 0, 7, 14, 21 and 28 d, respectively. A total of 535 metabolites were obtained and devided into 11 groups including flavonoids, amino acid and its derivatives, lipids, organic acids and its derivates, nucleotide and its derivates, alkaloids, hydroxycinnamoyl derivatives, carbohydrates and alcohols, phytohormones, coumarins and vitamins. Totally, 118 differential metabolites (VIP ≥ 1, *P* < 0.05) during chilling treatment process were detected, and their KEGG pathways involved in several metabolic pathways related to dormancy. Sucrose was the most abundant carbohydrate in peony bud. Starch was degradation and Embden Meyerhof Parnas (EMP) activity were increased during the dormancy release process, according to the variations of sugar contents, related enzyme activities and key genes expression. Flavonoids synthesis and accumulation were also promoted by prolonged chilling. Moreover, the variations of phytohormones (salicylic acid, jasmonic acid, abscisic acid, and indole-3-acetic acid) indicated they played different roles in dormancy transition.

**Conclusion:**

Our study suggested that starch degradation, EMP activation, and flavonoids accumulation were crucial and associated with bud dormancy transition in tree peony.

## Background

The bud dormancy of woody plants is a complex process that allows plants to survive in harsh environments such as cold and drought, and it is classified as paradormancy, endodormancy, and ecodormancy [1], of which endodormancy is regulated by internal factors [2]. Endodormancy also has an important impact on the maintenence and producction of the plant, for instance, the anti-season cultivation of some fruit trees. Thus, much attention has been attracted to understand the mechanism of dormancy regulation. External environmental factors, short-day and low temperature, play an essential role in endodormancy induction, and sufficient low temperature accumulation is a necessary prerequisite to bud break [3–5]. In *Paeonia lactiflora*, low temperature accumulation at 5 °C for nine weeks was required to ensure the re-growth of buds [6]. Also, the buds of tree peony ‘Luhehong’ need to undergo 21 d chilling at 0–4 °C to ensure the following normal development at growth condition [7]. In addition, the requirements of chilling unit are in a genotype dependent manner [8], and significantly different among apical buds, lateral buds and catkins in Persian walnut (*Juglans regia* L.) [9].

Many studies have been performed on the mechanism of bud dormancy release, such as genomics, transcriptomics, proteomics and molecular biological analysis. These results indicated that vast changes in the metabolism including carbohydrate inter-conversion and transport, lipid mobilization, nitrogen metabolism, phytohormone metabolism and redox processes are associated with chilling induced buds dormancy break [8, 10–13]. Till now, there is still rare study on the systematic changes of metabolites and their crosslinks during chilling induced dodormancy transition.

Carbohydrates play multiple roles in plant growth and development, besides acting as the primary source of carbon and energy. Before oxidative phosphorylation, Embden Meyerhof Parnas (EMP), Tricarboxylic Acid (TCA), and Pentose Phosphate Pathway (PPP) are the main respiration pathways in plants. EMP starts from glucose, which is an end product of starch degradation. Additionally, maltose and fructose also involve in the EMP pathway after conversion to glucose. The anabolic metabolism of sucrose is mainly carried out by two enzymes, sucrose synthase (SUS; EC 2.4.1.13) and invertase (INV; EC3.2.1.26) [14]. SUS reversibly catalyzes the formation of sucrose from UDP-glucose and fructose [15], and INV, which irreversibly decompose sucroses into hexose, can be divided into three categories: cell-wall invertase (CWIN), vacuolar invertase (VIN), and cytoplasmic invertase (CIN) [16, 17].

It had been reported that EMP, TCA, and PPP are strictly related to dormancy release in different plants. For example, the TCA cycle is enhanced, while the PPP pathway slowly decreased during apple bud sprouting [18]. In grape, dormancy release induced by chemical and low temperature was found related to PPP, EMP, and TCA cycles [19–21]. Furthermore, carbohydrates could also act as a sugar signaling molecule. Mason et al. (2014) found that sucrose could serve as a signaling molecule involved in paradormancy release [22].

Flavonoids are widespread secondary metabolites in plants, which mainly contain six subclasses: chalcones, flavones, flavonols, flavandiols, anthocyanins, and proanthocyanidins or condensed tannins [23]. The pathway of flavonoid biosynthesis is quite conservative and well understood in some model plants [23]. Some genes involved in the production of common precursors, such as *chalcone synthase* (*CHS*), *chalcone isomerase* (*CHI*), *flavanone 3-hydroxylase* (*F3H*), and *flavonoid 3′-hydroxylase* (*F3’H*)*,* are called Early Biosynthetic genes (EBGs). Correspondingly, downstream genes for flavonoid biosynthesis are called Late Biosynthetic genes (LBGs). The pathway of flavonoids biosynthesis can be affected by biotic and abiotic factors (e.g., pathogen infections, temperature, drought, plant hormones) [24]. Moreover, recent reports had shown that flavonoids involved in plant stress response [25], pollen development [26], color formation [27], etc. However, it is still unknown whether flavonoids participate in the process of dormancy release.

It is well known that external factors always work through internal factors during bud dormancy release, and hormones play an important role in dormancy regulation. With the extension exposure to dormancy-inducing conditions (short-day or low temperature), the expression of growth-promoting signals gene (*FLOWERING LOCUS T*, *FT*) is inhibited, leading to reduce gibberellins (GAs) levels and increase abscisic acid (ABA) contents, and the ABA response could induce the close of plasmodesmata, thereby mediating the establishment of dormancy [28, 29]. GA and ABA not only involve in the establishment of growth cessation, but also play a key role in dormancy release. During dormancy release, the reopening of plasmodesmata could restore the supply of growth-promoting signals with the increasing biosynthesis of GAs [30]. On the other hand, the degradation of ABA is necessary for bud dormancy release in grapes, while ABA is accumulated during dormancy establishment [31]. The recent reports showed that *SHORT VEGETATIVE PHASE (SVP)-like* (*SVL*) with sequence homology to the *Dormancy Associated MADS-box* (*DAM*) genes [32], plays a vital role in the dormancy of poplar [33]. Low-temperature decreases ABA levels and reduces *SVL* expression, leading to the induction of *FT1* expression and GA biosynthesis, which promotes dormancy release.

Tree peony (*Paeonia suffruticosa* Andr.) belonging to the *Moutan* subfamily of the genus *Paeonia*, Paeoniaceae, is one of the most ancient ornamental and medicinal plants in the world. The bud of tree peony is a typical compound bud with scale forming in autumn [34], and it must undergo a period of low temperature to ensure the sprouting and flowering in the next year. Due to the short and concentrated florescence every year, its anti-season culture becomes an important content of the tree peony industry. Until now, the primary method of anti-season production is to provide sufficient low temperature exposure alone or chilling enduration combining with gibberellin application. Therefore, it is of great value to understand the mechanism of chilling induced dormancy release in tree peony. Our previous study characterized the relationship between chilling accumulation and dormancy status, the physiological status of tree peony ‘Luhehong’ after 14 d chilling treatment was regarded as the transition stage from endodormancy to endodormancy release, and that after 21 d chilling treatment was defined as dormancy release, and after 28 d chilling as a stage of ecodormancy [35]. GA pathway plays a crucial role in endodormancy release induced by chilling [36]. And the activity of PPP pathway also increases during the process, suggesting that it play a role in dormancy release of tree peony [7]. As known, traits are more closely related to metabolites, which may provide a new perspective for the understanding of dormancy transition in tree peony.

Here, metabolic changes of tree peony buds during the chilling induced dormancy transition were analyzed. Kyoto Encyclopedia of Genes and Genomes (KEGG) enrichment analysis showed that differential metabolites involved in various metabolic pathways such as carbon metabolism, secondary metabolite synthesis, and hormone metabolism. It was revealed that starch degradation and EMP activity were enhanced during dormancy release. Interestingly, flavonoids anabolism was also activated by chilling accumulation, and its increasement might in return promote flower bud development. Furthermore, the concentration of plant hormones, such as ABA, jasmonic acid (JA), salicylic acid (SA),and indole-3-acetic acid (IAA) during the dormancy transition were also evaluated in this research. Significantly, the roles of flavonoids were firstly discussed during the dormancy transition in perennial plants. All results would provide valuable information for the molecular mechanism of dormancy transition in tree peony.

## Results

### Metabolomics analysis during chilling induced dormancy transition

**Fig. 1:**
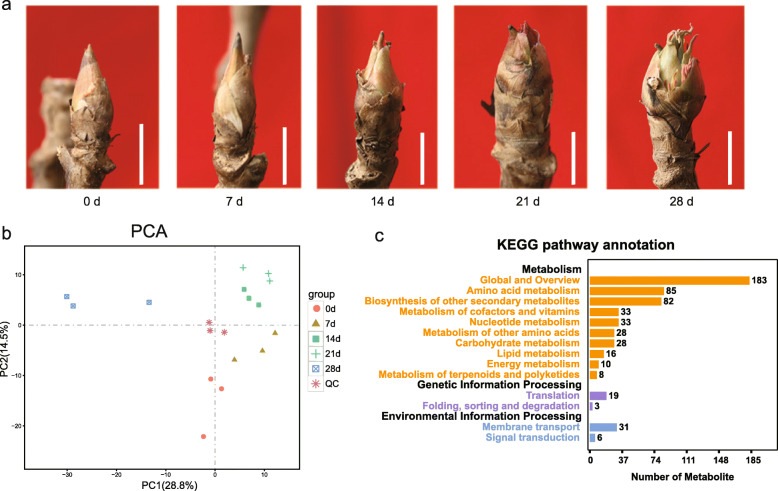
Metabolomics analysis of tree peony buds. **a** Bud morphology of tree peony buds during the chilling duration. The scale bar indicated 1 cm. **b** PCA plot of the tree peony buds at five stages. 0 d, 7 d, 14 d, 21 d and 28 d indicated days for chilling duration. QC, quality control samples. **c** The KEGG pathway annotation of metabolites. Black texts indicated the first hierarchy of KEGG Pathway database, and colored texts indicated the second hierarchy of KEGG Pathway database. The global and overview is a secondary metabolism name, containing eight metabolic pathways: Metabolic pathways, Biosynthesis of secondary metabolites, Microbial metabolism in diverse environments, Carbon metabolism, 2-Oxocarboxylic acid metabolism, Fatty acid metabolism, Biosynthesis of amino acids, Degradation of aromatic compounds

To study the metabolic changes during chilling-induced dormancy release in tree peony, flower buds were picked at five time points after 0–4 °C treatment for 0, 7, 14, 21 and 28 d, respectively, and metabolic profiling was analyzed by ultra-performance liquid chromatography (UPLC) and tandem mass spectrometry (MS/MS) (Fig. 1a). The Principal Component Analysis (PCA) analysis was employed to evaluate the repeatability of the metabolite profiles (Fig. 1b). As shown, quality control (QC) samples were separated from tested samples, and the two principal components (PC) accounted for 28.8% (PC1) and 14.5% (PC2) of the total variance, respectively. The samples were clearly separated into the 5 different subgroups by PC1 and PC2 (Fig. 1b).

A total of 535 metabolites were detected in the metabolomic analysis, and 511 of them were annotated with MassBank, KNAPSAcK, HMDB [37], MoTo DB and METLIN [38] (Table S1). The metabolites were divided into eleven groups, including flavonoids, amino acid and its derivatives, lipids, organic acids and its derivates, nucleotide and its derivates, alkaloids, hydroxycinnamoyl derivatives, carbohydrates and alcohols, phytohormones, coumarins, and vitamins (Table 1). Notebly, 123 flavonoids and derivates and 15 phytohormones were annotated (Table S1), which benifited to analyze their function. The results of metabolic pathway analysis showed that they were mapped to 14 KEGG pathways (Fig. 1c), of them, totally 183 metabolites were assigned to the pathway of global and overview, followed by amino acid metabolism with 85, biosynthesis of other secondary metabolites numbered 82, metabolism of cofactors and vitamins numbered 33, nucleotide metabolism, carbohydrate metabolism amounted 33, and so on (Fig. 1c).

**Table 1:** Overview of annotated metabolites

Type	Number	Percentage (%)
Flavonoids	132	25.83
Amino acid and its derivatives	79	15.46
Lipids	64	12.52
Organic acids and its derivates	62	12.13
Nucleotide and its derivates	51	9.98
Alkaloids	36	7.05
Hydroxycinnamoyl derivatives	27	5.28
Carbohydrates and alcohols	21	4.11
Phytohormones	14	2.74
Coumarins	9	1.76
Vitamins	9	1.76

### Differential metabolites analysis

**Fig. 2:**
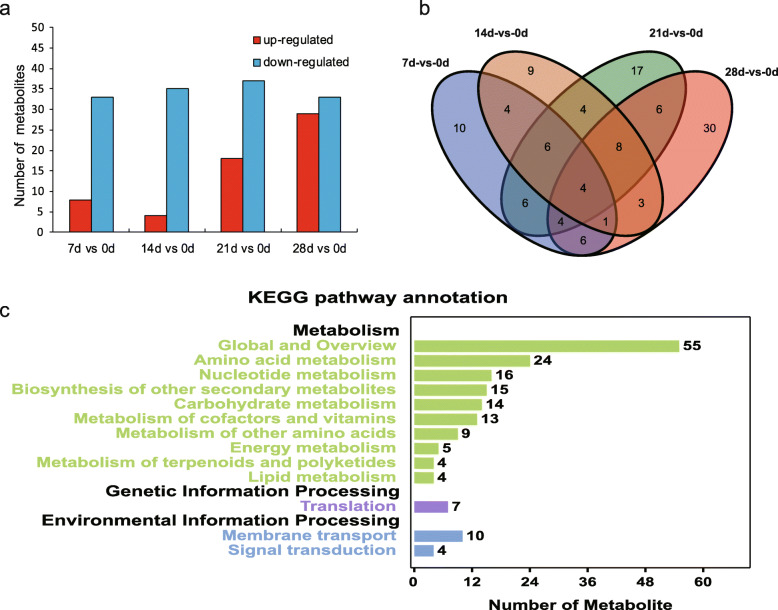
Expression dynamics and comparative analysis of metabolites in tree peony buds chilled at different stages. **a** Statistical map of intergroup metabolites (*P* < 0.05, VIP ≥ 1). Red and blue represented up- and down-regulated metabolites, respectively. **b** Venn diagram of metabolites among four comparison groups. **c** The KEGG pathway annotation of all DMs

The Orthogonal Projection to Latent Structures-Discriminant Analysis (OPLS-DA, VIP ≥ 1) and the Student’s t test (*P* < 0.05) were applied to detect the differential metabolites (DMs) among different treatments (Fig. S1). Totally, 118 DMs were obtained when the chilling treated groups were compared with that of 0 d samples. The results also indicated that the amounts of up-regulated metabolites increased along with the increase of chilling days (Fig. 2a). In detail, the number of up-regulated metabolites were 4, 18, and 29 when 14 d vs 0 d, 21 d vs 0 d, and 28 d vs 0 d, respectively. There were not regular trend for the number of down-regulated metabolites when compared with 0 d. The results indicated that some metabolism pathways were activated by the prolonged chilling exposure. Interestingly, the number of up-regulated metabolites decreased when comparing 14 d vs 0 d with 7 d vs 0 d. To better understand the metabolites changes caused by different chilling durations, the venn diagram of 118 DMs was constructed, showing that four metabolites were common among the four comparisons, i.e. 7 d vs 0 d, 14 d vs 0 d, 21 d vs 0 d, and 28 d vs 0 d (Fig. 2b; Fig. S2). Besides, 30 metabolites were unique in 28 d vs 0 d, implying that wider changes happened in this group. The KEGG pathway annotation of 118 DMs was performed and the DMs involved in amino acid metabolism (24 in number), nucleotide metabolism (16), and biosynthesis of other secondary metabolism (15), and so on (Fig. 2c). Additionally, KEGG enrichments between each two treatments were listed in Fig. S3. Carbohydrate metabolisms, such as pyruvate metabolism, starch and sucrose metabolism, PPP pathway and so on, were frequently presented in the different enrichments analysis. Plant hormone signaling transduction was also enriched in 7 comparable groups. Besides above, several amino acid metabolisms, pyrimidine and purine metabolism, nitrogen metabolism and others were also frequently enriched in the comparations (Fig. S3).

### The metabolic processes related to dormancy release in tree peony

**Fig. 3:**
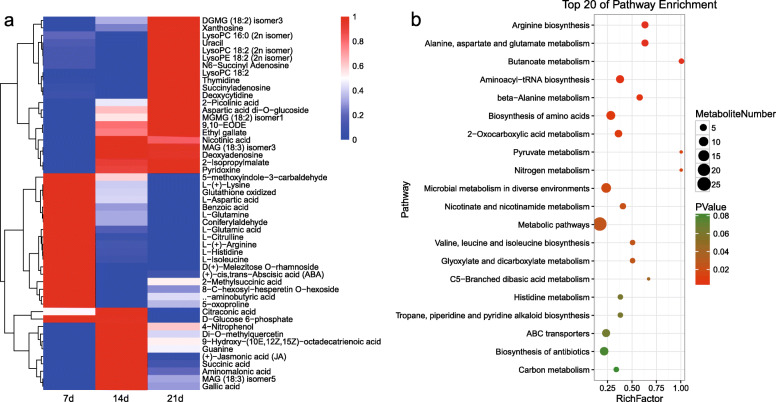
Changes of 50 DMs related to dormancy release in tree peony. **a** The clustering heatmap of 50 DMs at 14 d vs 7 d and 21 d vs 7 d. **b** The top 20 of KEGG enrichment pathways for DMs among 14 d vs 7 d and 21 d vs 7 d

To study the crucial metabolic processes related to endodormancy release in tree peony, DMs of 14 d vs 7d and 21 d vs 7 d were screened. Totally, 50 DMs were obtained and presented in a clustering heatmap, which showed the metabolites change from the dormancy to dormancy release (Fig. 3a). Among them, 21 DMs were significantly up-regulated, 18 DMs were down-regulated with the prolonged chilling enduration, and the other 11 DMs fluctuated with a peak at 14 d. The KEGG analysis of the 50 DMs showed that 25 DMs participated in the metabolic pathway, which accounted for 83.33% of all the 30 annotated metabolites (Fig. 3b, Table S2). The KEGG enrichment anaylsis of 25 DMs showed that they involved in the pathways of glucose metabolism (glucose 6-phosphate), amino acid metabolism (aspartate) and hormone metabolism (ABA) (Table S3), suggesting that these metabolic pathways might play a critical role during dormancy release in tree peony.

### Carbon metabolism during dormancy transition in tree peony

**Fig. 4:**
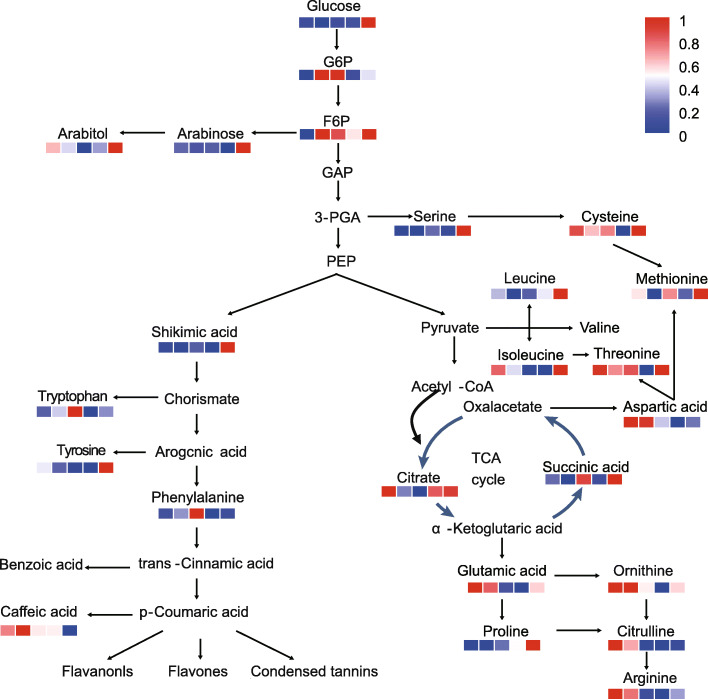
Dynamics of carbon metabolic pathways throughout the chilling duration process. The metabolite amounts were shown in heatmaps as 0, 7, 14, 21, and 28 d from left to right, respectively. G6P (glucose 6-phosphate), F6P (fructose 6-phosphate), GAP (3-phosphoglyceraldehyde), PEP (phosphoenolpyruvate)

A metabolic network containing EMP pathway, TCA cycle, shikimate pathway, and amino acid metabolism was presented to visualize the carbon flow during dormancy release of tree peony (Fig. 4). The levels of glucose at 0, 7, 14 and 21 d were lower than that at 28 d. However, glucose 6-phosphate (G6P) and fructose 6-phosphate (F6P) had the higher levels at 7 and 14 d (Fig. 4), indicating that the EMP was activated during chilling induced endodormancy release in tree peony. In the TCA cycle, the levels of citrate declined until 14 d and then climbed. The succinic acid amount showed an significant upward at 14 d. In terms of amino acid metabolism, some amino acids increased with the release of endodormancy, such as leucine, proline, etc., while some others decreased, such as valine, aspartic acid, glutamic acid, etc. (Fig. 4). In the shikimic acid pathway, the level of shikimic acid and phenylalanine increased after chilling exposure (Fig. 4).

### The variation of carbohydrates during dormancy transition

**Fig. 5:**
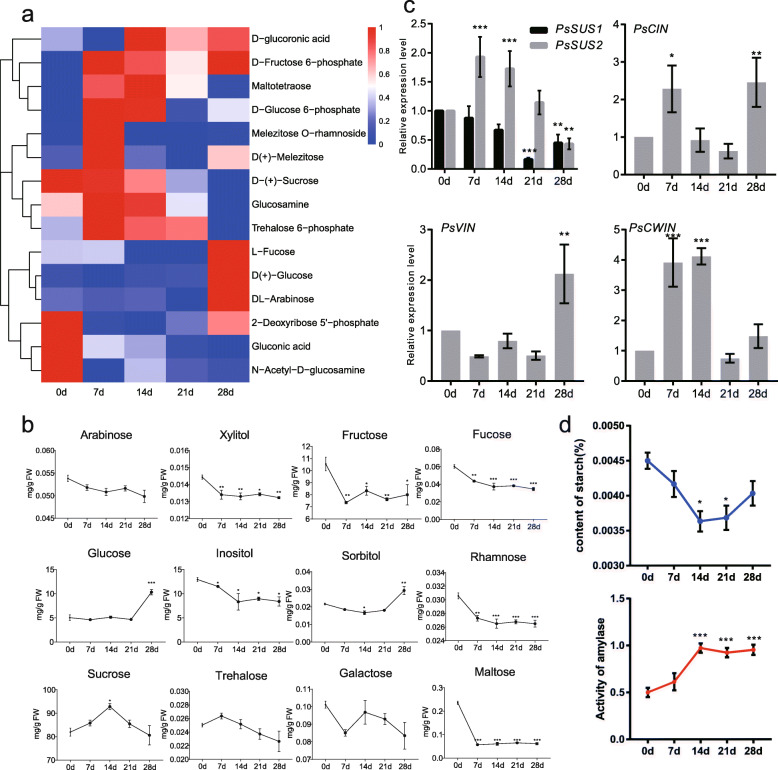
Changes of carbohydrates during dormancy transition induced by the chilling in tree peony. **a** The cluster heatmap of carbohydrates. **b** The variations of several sugars content. **c** The relative expression levels of sucrose synthase and sucrose invertase genes. **d** The variations of starch and amylases activity. The mean ± SD in three biological replicates was shown. *, ** and *** indicated significant differences of one-way ANOVA at *P* < 0.05, *P* < 0.01, and *P* < 0.001, respectively

Sugars play crutial roles in energy metabolism and substance metabolism, whose variation might reflect the status of bud dormancy after chilling exposure. The levels of 15 sugars in metabolic profiles were analyzed throughout the dormancy process (Fig. 5a). Some monosaccharide (G6P, glucosamine, and trehalose 6-phosphate) and polysaccharides (maltotetraose, melezitose, and melezitose *O*-rhamnoside) were up-regulated after 7 d chilling exposure, and then their contents reduced (Fig. 5a). The monosaccharides (fucose, glucose, and arabinose) had the maximum level at the ecodormancy stage (28 d chilling treated, Fig. 5a), indicating a well preparatory status for the following re-growth. A Gas Chromatography-Tandem Mass Spectrometry (GC-MS/MS) measurement was then employed to further analyze the change of sugars during the chilling process in tree peony, and the results were similar to the metabolomics data. Fructose, glucose, and inositol were the three most abundant kinds of monosaccharides. The content of maltose significantly decreased after 7 d chilling treatment. Remarkably, the content of sucrose was the hightest of all the 13 tested sugars, which significantly increased and reached a maximum of 92.9 mg/g at 14 d chilling, and then declined rapidly (Fig. 5b), suggesting that it might play a vital role in the whole process. To further investigate the role of sucrose in dormancy release of buds, the expression patterns of *sucrose synthase* (*PsSUS1* and *PsSUS2*) and *sucrose invertase* genes were analyzed (Fig. 5c, Fig. S4). The expression of *PsSUS1* continued to decline after 7 d chilling treatment, but *PsSUS2* was up-regulated at 7 and 14 d chilling. The expressions of *cytoplasmic invertase* (*PsCIN*), *vacuolar invertase* (*PsVIN*), and *cell-wall invertase* (*PsCWIN*) were significantly increased at different chilling periods (Fig. 5c). Taken together, it was presumed that sucrose catabolism was dominant during chilling duration process, to provide sufficient sugars for respiratory metabolism and energy metabolism. Starch, the main storage carbohydrate in higher plants, was measured during dormancy transition in tree peony. The results indicated that starch content decreased after chilling exposure and reached its minimum at 14 d, which might be related to the activity of amylases (AMY) during the same process (Fig. 5d).

### The changes of flavonoids during dormancy transition

**Fig. 6:**
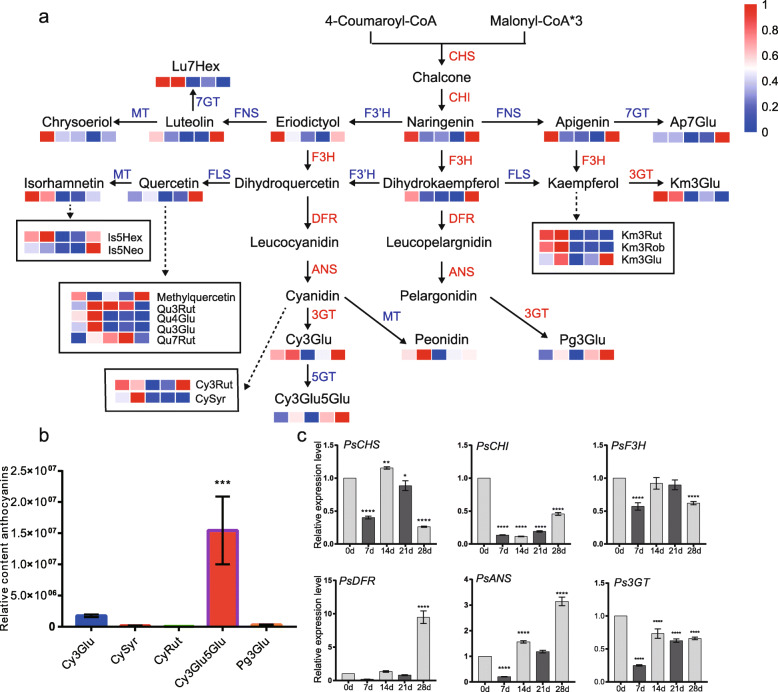
The changes of flavonoids content and the related genes expression during the chilling duration. **a** The flavonoids and anthocyanin pathway. CHS, chalcone synthase; CHI, chalcone isomerase; F3H, flavanone 3-hydroxylase; DFR, dihydroflavonol 4-reductase; ANS, anthocyanin synthase; 3GT, anthocyanidin 3-*O*-glycosyltransferase; 5GT, anthocyanidin 5-*O*-glycosyltransferase; MT, anthocyanidin methyltransferase; 7GT, anthocyanidin 7-*O*-glycosyltransferase; FLS, flavonol synthase; FNS, flavone synthase; Lu, luteolin; Ap, apigenin; Is, isorhamnetin; Qu, quercetin; Km, kaempferol; Cy, cyanidin; Pg, pelargonidin; Glu, glucoside; Hex, hexoside; Neo, neohesperidoside; Rut, rutinoside; Rob, robinobioside; Syr, syringic acid. **b** The relative content of anthocyanins in tree peony flower buds after 28 d chilling duration based on the results of LC-ESI-MS/MS. **c** The relative expression levels of key genes involving in anthocyanin biosynthesis. Data were represented as mean of three different determinations ± SD. Asterisks indicate statistically significant differences (one-way ANOVA, *P < 0.05, **P < 0.01, ***P < 0.001 and ****P < 0.0001)

In KEGG enriched terms, the biosynthesis of secondary metabolites accounted for a large part (Fig. 3b), of which flavonoids are secondary metabolites that are widely present in plants. Thus a flavonoids metabolic pathway analysis was performed to understand its changes during dormancy transition in buds. Most flavonoids (naringenin, apigenin, luteolin, etc.), flavonols (kaempferol, quercetin, etc.), and anthocyanins (cyanidin 3-*O*-glucoside, pelargonidin 3-*O*-glucoside, etc.) showed similar down-regulated tendency at the early stages of dormancy (0–14 d) and up-regulated at 28 d. Thus, their highest contents were usually detected at 28 d, such as cyanidin-base anthocyanins, cyanidin 3-*O*-glucoside etc. (Fig. 6a). According to the results of LC-ESI-MS/MS, the relative contents of anthocyanins were analyzed at 28 d. The results indicated that five anthocyanins, including cyanidin 3-*O*-glucoside (Cy3Glu), cyanidin 3-*O*-rutinoside (CyRut), cyanidin *O*-syringic acid (CySyr), cyanidin 3,5-di-*O*-glucoside (Cy3Glu5Glu), and pelargonidin 3-*O*-glucoside (Pg3Glu), were dominant in flower buds of tree peony, and the others were relactively rare (Fig. 6a and b). In detail, cyanidin-base anthocyanins (Cy3Glu5Glu) accounted for the majority, followed by Cy3Glu (Fig. 6a and b). Two *EBGs* (*PsCHS* and *PsF3H*) and two *LBGs* (*PsANS* and *Ps3GT*) showed the similar patterns excluding *PsDFR*. Their transcripts were relactively abundant at the beginning of dormancy (0 d), and dramatically declined at 7 d chilling, but prolonged chilling promoted their expression compared to 7 d treatment. The *PsDFR* remained very low expression level till dormancy release period, but significally increased by ten folds at 28 d comparing with 0 d (Fig. 6c, Fig. S4). The expression patterns of the raltaed genes were accordance with their content variations during chilling duration process.

### The variation of phytohormones during dormancy transition

**Fig. 7:**
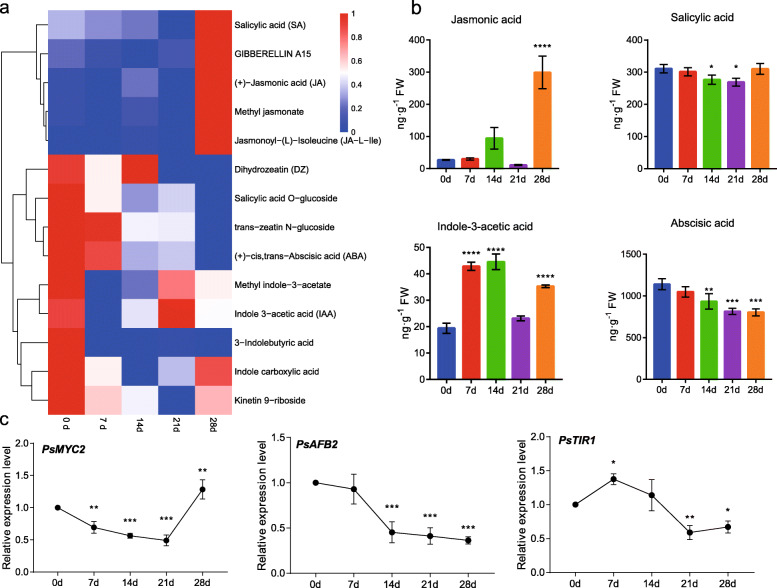
The changes of phytohormone contents and the expression patterns of related genes during chilling duration. **a** The cluster heatmap of phytohormone during the chilling duration. **b** Phytohormone contents in tree peony flower buds during the chilling duration by LC-MS/MS. **c** The qRT-PCR results of JA related gene *PsMYC2*, and two auxin receptors genes *PsAFBs*, and *PsTIR1.* The mean ± SD (*n* = 3) were shown. Asterisks indicated statistically significant differences (one-way ANOVA, *P < 0.05, **P < 0.01, ***P < 0.001 and ****P < 0.0001)

Phytohormones play vital roles in plant growth and development, flowering, stress response, and so on. In metabolomics analysis, 14 phytohormones or analogues, including SA, JA, GA, and ABA, were detected in dormant buds of tree peony, and their content variations during chilling duration were shown in a heatmap (Fig. 7a). Nine metabolites presented the highest contents at chilled 0 d (nonchilled period), and the other five hormones peaked at chilled 28 d (Fig. 7a). They were divided into three subgroups by cluster analysis The first subgroup showed a up-regulated tendency with a peak at 28 d, including SA, GA_15_, JA, methyl jasmonate (MeJA) and jasmonic acid-isoleucine (JA-Ile). Dihydrozeatin, salicylic acid *O*-glucoside, trans-zeatin *N*-glucoside and ABA were categorized into the second subgroup with an obviously down-regulated tendency. The others fluctuated during chilling exposure process with a peak at 0 d chilling point.

The contents of JA, SA, IAA and ABA were also evaluated by LC-MS/MS analysis, and the results were similar to the metabolomics data. JA levels were relatively low from 0 to 21 d chilling, and dramatically peaked to 299.167 ng/g with a ten folds increase at chilled 28 d (Fig. 7b). The MYC2 (myelocytomatosis protein 2) transcription factor plays a central role in JA signal transduction [39]. Consistent with the variation of JA contents, the transcript of *PsMYC2* increased sharply at chilled 28 d (Fig. 7c, Fig. S4). The contents of SA were down-regulated from 0 d to 21 d, and then recovered the initial content of 0 d. The contents of IAA fluctuated during the chilling duration process, reached the highest level of 44.57 ng/g at 14 d, and its content of 28 d was also higher than 0 and 21 d (Fig. 7b). The expression pattern of two auxin receptors genes (*TRANSPORT INHIBITOR RESPONSE1/AUXIN SIGNALING F-BOX PROTEIN2,TIR1/AFB2*) were analyzed during the chilling duration. *PsAFB2* was down-regulated in the whole process, and *PsTIR1* was up-regulated at 7 d, and then down-regulated from 7 to 28 d (Fig. 7c, Fig. S4). The contents of ABA persistently decreased from 1140.37 ng/g at 0 d to 803.83 ng/g at 28 d (Fig. 7b), indicating that ABA was an inhibitor of dormancy release in tree peony buds, and chilling treatment could gradually reduce the contents of ABA.

## Discussion

The dormancy transition of woody plants is a complicated process, which was synergetically regulated by photoperiod and low temperature [40]. Chilling treatment as an effective method to promote dormancy release had been verified in many species including tree peony [3–5, 9, 41]. Transcriptomics, proteomics and microRNAs analysis had been performed to investigated the complex mechanism of chilling induced dormancy release in tree peony, which revealed the roles of GA and carbohydrate metabolism [7, 13, 27]. Here, substance changes were detected by metabolomics analysis during chilling induced dormancy transition, and the expression of related genes were also analyzed by qRT-PCR. Several metabolsim pathways were enriched and 118 metabolites were differential in the process..

### Starch degradation and EMP pathway were enhanced during dormancy release

Carbohydrate is the basic energy substance of primary metabolism and secondary metabolism. Sugars metabolism and signal transduction involving in bud dormancy process had been revealed by RNA-seq [42]. Sugar metabolism genes (*beta-amylase 5,alpha-amylase-like 1, sucrose synthase 3,* and *trehalose-phosphatase/synthase 7*), sugar transporter genes (*GT-2 like 1, sucrose-proton symporter 2, protein O-mannosyltransferase 5,* and *senescence-associated gene 29*) and sugar signal transduction genes (*glucose insensitive 2* and *beta-fruct 4*) had been proven to involve in dormancy release in poplar, grape and *P. mume* [42–44]. In this study, the contents of carbohydrates were continuously measured during the chilling induced dormancy transition of tree peony. Both starch and maltose showed downward trend before dormancy release, which were consistent with the increasing of amylase activity and sugar catabolism (Fig. 5b, d). At the same time, the enzyme activity of AMY gradually increased. Our recent results also revealed that the transcripts of *PsAMY* and *PsBMY* were up-regulated during the same process [7, 8, 42]. Together, these results indicated that chilling treatment promoted the degradation of starch during dormancy release in tree peony. After endodormancy release (21–28 d), the buds maintained active respiratory metabolism and amylase activity, but the contents of starch and maltose did not decrease significantly (Fig. 5b, d). It was speculated that a considerable amount of carbohydrates were transported from other parts to the buds, such as the root system (tree peony has a developed succulent root system), to meet the consumption of flower buds. In addition, the reopening of the material transport channel might be another important reason, which enabled the long-distance transport of carbohydrates [30].

In our study, starch was rapidly degraded at the beginning of the chilling duration due to high amylase activity, and sucrose was a kind of its intermediate product. The content of sucrose was the most abundant among the measured sugars, which implied that sucrose might also be used to transport of assimilates in tree peony rather than sorbitol as in apple because sucrose content was about 3200 folds higher than that of sorbitol. Sucrose was up-regulated till to 14 d and decreased afterward according to GC-MS/MS results (Fig. 5b), although sucrose synthase gene *SUS1* was upregulated by chilling duration. Our results were different to that in Persian walnut with a persistent increase of sucrose [8]. Taken together, we speculated that sucrose mainly came from starch degradation, and were rapidly utilized for the following EMP pathway and so on. It was also found that sucrose accumulated, and sucrose synthase genes were up-regulated at the early stage of dormancy process in poplar and *P. mume* [42]. Therefore, sucrose might be used as an energy center to ensure the supply of glucose in dormancy transition of tree peony.

In addition, extensive degradation of starch should lead to glucose accumulation, but glucose and fructose were at a low level or down-regulated until the ecodormancy stage (Fig. 5a, b). Meanwhile, the contents of F6P and G6P increased significantly after chilling exposure, and thereafter decreased (Fig. 4 and 5a). Also, Our previous study showed that the transcripts (*HK* and *G6P*) and enzyme activities of Hexokinase and glucose 6-phosphate isomerase were also significantly up-regulated [7, 42]. These results indicated that the EMP pathway was activated after chilling exposure in tree peony. Metabolomics results showed that dormancy release was an energy-consuming process. A large number of carbohydrates were broken down to produce enough substances and energy to promote dormancy release, which also providied a carbon chain for secondary metabolism.

### Flavonoids accumulation at ecodormancy stage

As secondary metabolites, flavonoids play important roles in many processes of plant growth and development, such as color formation, stress resistance, etc. [15]. However, the changes and functions of flavonoids during dormancy transition were still poorly understood. Variations of flavonoids were analyzed during chilling duration process in our work. Flavonoids (e.g. quercetin, kaempferol, and apigenin) were synthesized in the early step of the flavonoid biosynthesis pathway, which were down-regulated in the whole endodormancy stage and sharply upregulated at 28 d, corresponding to the decreasing expression of *PsCHI* (Fig. 6). The flavonoids accumulation at early stage of chilling treatment (0 d and 7 d) might be related to cold tolerance for winter survival. It was considered that the initial products of the flavonoids biosynthesis pathway, such as quercetin, kaempferol, apigenin, etc., inhibited the transport of polar auxin to regulate plant development [45]. Also, the correlation between pollen fertility and flavonoids have been found in maize and peanuts [23], and the silencing of *chalcone synthase* gene resultes in parthenocarpy in tomato [46]. Flavonols (in particular quercetin) is essential for pollen germination in tobacco [26]. In our results, flavonols showed higher levels at ecodormancy stage (Fig. 6a), when the flower buds were well-differentiated, and stamens were clearly visible [34]. Therefore, it was speculated that flavonols might be involved in flower bud development at ecodormancy stage in tree peony.

In a recent study, Gu et al. (2019) suggested that anthocyanin accumulation occurred 10 d before anthesis in tree peony ‘Qing Hai Hu Yin Bo’ [47]. Here, we found anthocyanins were up-regulated after endodormancy release, accumulating in large amounts at ecodormancy stage in tree peony ‘Luhehong’ (Fig. 6). The results implied that the floral pigments might begin their synthesis and accumulation before the bud entering into endodormancy with a peak at ecodormancy period. Cyanidin-based glycosides such as Cy3Glu and Cy3Glu5Glu were the most abundant anthocyanins in the petal blotches of 35 cultivars [48], and they were also the most abundant anthocyanins in the buds of ‘Luhehong’ (Fig. 6b). We hypothesized that chilling-induced dormancy release synchronously activated anthocyanin synthesis and accumulation.

Taken together, flavonoids synthesis and accumulation could be actived by prolonged chilling, and dormancy release might accelerate flower bud development through flavonoids accumulation. To our knowledge, it was the first report to describe the changes and role of flavonoids during dormancy transition in perennial woody plants.

### The roles of phytohormone during dormancy transition

In perennial woody plants, ABA and GA have been widely proven to regulate bud dormancy. Recent researches revealed the antagnism between ABA and GA in bud dormancy. The GA and ABA pathways are found to be the most enriched in the comparison of different dormancy stages in grape [49]. A MADS-box (DAM) family gene, *SHORT VEGETATIVE PHASE-like (SVL)* have been found to play a key role in ABA-mediated bud dormancy in poplar. In *SVL* RNAi strain, the expression of *FT1* is significantly up-regulated, and GA biosynthesis key enzyme gene *GA20ox* is up-regulated to promote bud rupture [32]. Further research found that SVL could directly bind to the promoters of *GA2ox8* and *CALS1* to induce their expression, reduce the levels of active GAs and control the closure of plasmodesmata, thereby maintain dormancy status [50]. Chilling accumulation reduces the level of ABA, which in turn suppresses the expression of *SVL* and promotes the biosynthesis of GAs, and finally break bud dormancy [36]. In our work, ABA was persistently down-regulated along with the chilling duration (Fig. 7), which was similar to grape during domancy transition process [51]. Gibberellin 15, the precursor of bioactive gibberellins, was up-regulated gradually (Fig. 7a). Meanwhile, the expression of *PsGA20ox* was up-regulated, and the content of GAs increased with chilling accumulation in tree peony [51, 52]. Therefore, there might be similar mechanisms between ABA and GA-regulated dormancy in poplar and tree peony.

Auxins and cytokinins (CKs) play antagonistic roles in meristems of many plants [53]. Previous researches had shown that CKs play a positive role in hydrogen cyanamide-induced bud dormancy release in grape [54], but that of IAA is still ambiguous until now. In our study, the level of CKs (dihydrozeatin, *trans*-zeatin *N*-glucoside and kinetin *9*-rboside) decreased after chilling exposure, and IAA was up-regulated by chilling through metabolomics and LC-MS/MS analysis (Fig. 7). TRANSPORT INHIBITOR RESPONSE1/AUXIN SIGNALING F-BOX PROTEIN (TIR1/AFB) family are known as auxin receptors [55]. The transcripts of *PsTIR1* were significantly induced and consistent with the IAA contents variation (Fig. 7c), which implied a positive role of IAA during dormancy release in tree peony. These results were different from the upregulation of CKs in *P. kingianum* and *P. mume* during the same process [56, 57]. Therefore, the regulations of CKs and IAA in dormancy release were not a common mechanism in different perennial plants, and they might not be the key factors in dormancy regulation.

Usually, SA and JA are regarded as stress response hormones, rather than function on dormancy regulation [58, 59]. Recently, Ionescu et al. (2017) proposed that the upregulation of JA-Ile induced the expression of *MYB21* (*myeloblastosis viral oncogene homolog B21*) and *MYB108* to participate in the flower development process during HC-induced dormancy release in sweet cherry [57]. In our results, JAs were dramatically upregulated when entering ecodormancy period, along with a transcriptional increase of *PsMYC2*, a key transcription factor for JA signal transduction (Fig. 7). The results indicated that JA signal transduction was activated after the fufilment of chilling accumulation. In a recent study, the mechanism of JA promoting the anthocyanins accumulation was revealed in *Arabidopsis*. When the JA content increases, the inhibitory effect of JAZs (jasmonate ZIM-domain) protein on MYB-bHLH-WD40 complex is released, which will promote the expression of *DFR* and *ANS*, and the accumulation of anthocyanins [60]. Interestingly, *PsDFR* and *PsANS* also significantly increased, and anthocyanins presented higher levels at 28 d chilling treatment in our study (Fig. 7). It was hypothesized that JA additionally involved in anthocyanin accumulation after endodormancy release in tree peony. Additionally, SA involves in the response to low-temperature stress, for SA and glucosyl SA accumulating after low-temperature exposure [58]. The use of exogenous SA improves the cold tolerance of corn, cucumber, and rice [59]. The accumulations of SA and Salicylic acid *O*-glucoside were also observed during chilling induced dormancy release in tree peony (Fig. 7), it might be the response of buds to low-temperature stress.

## Conclusions

**Fig. 8:**
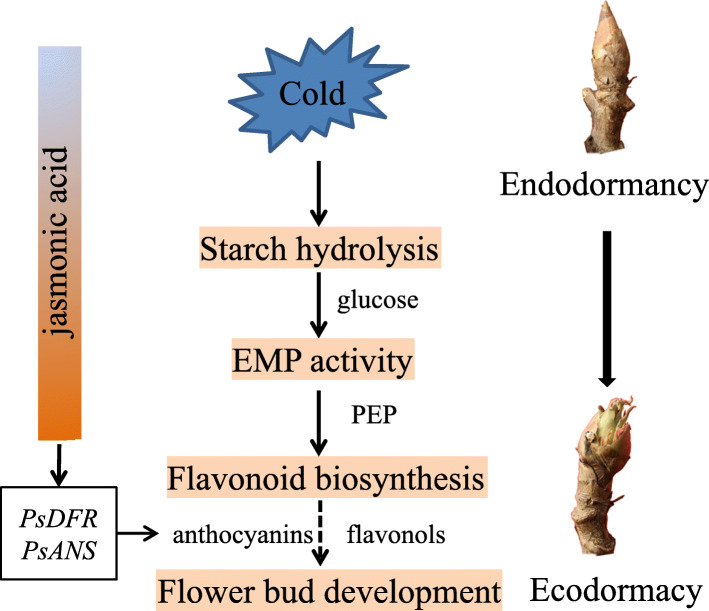
Model of metabolite role during dormancy transition induced by chilling in tree peony. Starch degradation and EMP activation provide energy and material basis for flavonoid accumulation during endodormancy release. Flavonoid and anthocyanin accumulation might promote flower bud development at ecodormancy stage. Meanwhile, the accumulation of anthoyanin may be regulated by JA

In summary, we systematically revealed the metabolomic changes during the chilling induced dormancy transition of tree peony, and a total of 511 substances and 118 DMs were identified. Chilling accumulation promoted the degradation of starch and enhanced the activity of EMP, providing adequate energy and substances for secondary metabolism required by dormancy release and bud burst. Flavonoid was accumulated by sufficient chilling duration along with endodormancy release. Furthermore, we also reported phytohormone changes during the dormancy transition in tree peony. Prolonged chilling exposure declined ABA content, but promoted JA and GA accumulation at the end of dormancy. Taken together, we proposed a work model of dormancy transition induced by chilling according to the metabonomics analysis (Fig. 8). Our results might help to better understand the dormancy transition of perennial plants.

## Materials and methods

### Plant materials

Four-years-old tree peony plants (*Paeonia suffruticosa* cv. Luhehong) were provided by Tree Peony Research Institute of Qingdao Agricultural University, Qingdao, China. The plants were treated with continuous artificial chilling (0–4 °C refrigeratory storage, dark/24 h) from Nov 12, 2018 with the daily average temperature less than 10 °C as described previously [7]. At 0, 7, 14, 21 and 28 d after refrigerating treatment, buds were picked and scales were removed in each time point, frozen in liquid nitrogen, and stored at − 80 °C until further analysis. The samples from each three plants were harvested and mixed in each treatment. Three replicates (3 plants/replicate) per group were set.

### Sample preparation and extraction

After freeze-drying, the samples were crushed at 30 Hz for 1.5 min using a mixing mill with zirconia beads (MM 400, Retsch). Then powder (100 mg) was mixed with 70% methanol solution (1 mL, containing 0.1 mg/L lidocaine as an internal standard) at 4 °C overnight. After centrifugation (10,000 g, 10 min), the supernatant was filtered (scaa-104, aperture 0.22 μm; ANPEL, Shanghai, China) and analyzed by LC-MS/MS. Quality control (QC) samples were mixed with all samples to test the repeatability of the whole experiment [61].

### Liquid chromatography electrospray ionisation tandem mass spectrometry (LC-ESI-MS/MS)

The extracted compounds were analyzed using an LC-ESI-MS/MS system (UPLC, Shim-pack UFLC SHIMADZU CBM20A; MS/MS, Applied Biosystems 4500 QTRAP) by Gene Denovo Biotechnology Co. Ltd., Guangzhou, China [62]. Firstly, the samples (5 μL) were added into a Waters ACQUITY UPLC HSS T3 C_18_ chromatographic column (2.1 mm × 100 mm, 1.8 m) with a flow rate of 0.4 mL/min at 40 °C. The water and acetonitrile were acidificated with 0.04% acetic acid, which were used as the mobile phases of stages A and B, respectively. The phase gradients to separate the compounds was as Chen et al. [62]. The Linear ion trap (LIT) and triple quadrupole (QQQ) scans were performed on the triple quadrupole linear ion TRAP mass spectrometer (QTRAP). Then AB Sciex QTRAP4500 (ABQ) system was equipped with an ESI-Turbo Ion-Spray interface. ABQ was ran in positive ion mode, and Analyst 1.6.1 software (AB Sciex) was used to operate according to Chen et al. [62].

### Qualitative and quantitative determination of metabolites

The qualitative analysis of metabolites was performed based on the public metabolite database (e.g. MassBank and KNApSAcK) and the METLIN database (MWDB) [37, 38]. The repetitive signal (e.g. K^+^, Na^+^, NH_4_^+^, and other large molecular weight species) were removed during the analysis process. The metabolites were quantified by multiple reaction monitoring (MRM) of triple quadrupole mass spectrometry. The total ion chromatogram (TIC) and extracted ion chromatogram (EIC or Xic) of QC samples were derived to summarize the metabolite spectra of all samples and calculate the area of each peak. The MultiaQuant software (v 3.0.3) was used to integrate and calibrate the peaks.

### Principal component analysis (PCA) and orthogonal projection to latent structures-discriminant analysis (OPLS-DA)

To initially visualize the differences between the groups, the R package “ropls” was employed for principal component analysis (PCA) (http://bioconductor.org/packages/release/bioc/html/ropls.html). Orthogonal projection to latent structures-discriminant analysis (OPLS-DA) is an development of PLS-DA, which comprises an Orthogonal Signal Correction (OSC) filter into a PLS model. The OPLS-DA model was used to analyze all comparison groups. And, subsequent model tests and differential metabolite screening were analyzed using OPLS-DA results.

### Differential metabolites analysis and KEGG analysis

The most distinguishable metabolites between every two groups were ranked by the variable importance of the projection (VIP) score according to OPLS model. The threshold for VIP was set to 1.0. Besides, Student’s t test was used as to screen differential metabolites. Those with *P* < 0.05 and VIP ≥ 1 were considered as differential metabolites between two groups. The KEGG Orthology software (http://kobas.cbi.pku.edu.cn/) was used for KEGG pathway analysis.

### RNA extraction and real time quantitative PCR analysis

After 0, 7, 14, 21 and 28 d 0–4 °C treatment, the total RNA was isolated from *P. suffruticosa* flower buds according to the protocol of RNA isolation kit (TaKaRa, Dalian, China). The DNase I (TaKaRa, Dalian, China) was used to remove genomic DNA. The first strand cDNA was synthesized by HiScript III RT SuperMix for qPCR (+gDNA wiper) (Vazyme, Nanjing, China). qRT-PCR was carried out using ChamQ Universal SYBR qPCR Master Mix (Vazyme, Nanjing, China) following the manufacturer’s protocol. The detailed reaction system and procedure were similar to the previous study [36]. Additionally, the *P. suffruticosa Actin* was used as an internal control to normalize the transcriptional levels based on our previous results of different candidate control genes (*Actin*, *beta-tubulin*, *alpha-tubulin* and *60S-L*_*11*_) [63]. The Primer Premier 6 was used to designed specific primers (Table S4), and the candidate genes were selected based on our previous transcriptional profile [36], with their phylogenetic trees presented in Fig. S4. The relative expression levels were calculated using 2^−ΔΔCt^ method [64].

### Measurements of sugar contents

Three flower buds of every repeat at five chilling points were crushed by a crusher (MM 400, Retsch) containing zirconia beads at 30 Hz. A total of 20 mg powder was added into 500 μL of methanol: isopropanol: water (3: 3: 2, V/V/V) solution. The supernatant (50 μL) were taken and evaporated in nitrogen after being vortexed (3 min) and sonicated (30 min), with adding internal standard. After evaporated in a nitrogen stream and freeze-drying, the residue was further derivated as follows: Firstly, the small molecule carbohydrate was mixed with methoxine hydrochloride solution (100 μL) in the 1.5 mL tubes. Secondly, bistrifluoroacetamide (100 μL) was added to the solution at 37 °C for 2 h. After vortexed, the mixture was incubated at 37 °C for 30 min. N-Hexane was used as dilution fusion. Then, mixture was detected by MetWare (http://www.metware.cn/) based on the Agilent7890B-7000D GC-MS/MS platform, and the parameters were set as reported by Gómez-González et al. [65] .

### Measurements of starch content and AMY enzyme activity

The contents of starch at five chilling points was determined according to the previous method with little change, respectively [66]. The crushed tree peony buds (0.1 g) were extracted in 7.2 mL of ethanol (80%) at 80 °C for 30 min. The extract was centrifuged for 30 min, and the precipitate was gelatinized in a boiling water bath for 15 min. Concentrated sulfuric acid was added to the precipitate to dehydrate monosaccharides into aldehyde compounds. The anthrone reagent was used to react with the test solution. The absorbance at 640 nm was recorded with a spectrophotometer (HITACHI, Japan).

The AMY enzyme activity was determined according to the method of Huggins and Russell (1948) with minor modifications [67]. About 0.2 g flower buds after different chilling fulfilling (0, 7, 14, 21 and 28 d) from tree peony was ground into a homogenate, and centrifuged to collect the supernatant, respectively. The supernatant was incubated at 70 °C for 30 min to inactivate β-amylase. The samples were reacted with 3,5-dinitrosalicylic acid. The absorbance at 525 nm was recorded with a spectrophotometer (HITACHI, Japan).

### Measurements of hormone contents

Approximately 2 g fresh weight buds per repeat were taken from different chilling treatments. The contents of phytohormones were determined by the Wuhan Greensword Creation Technology Company (http://www.greenswordcreation.com) based on LC-MS/MS analysis according to a previously reported method with minor modification [68].

### Statistical analysis

Means and standard errors were calculated using Graphpad Prism 7 (San Diego, USA). Analysis of variance (One way ANOVA) was used to compare statistical differences and levels of gene expression between treatments and control.

## Supplementary information

**Additional file 1: Table S1** Metabolites identified by LC-ESI-MS/MS and annotated.

**Additional file 2: Table S2.** KEGG pathway enrichment of dormancy release related DMs.

**Additional file 3: Table S3.** DMs related to dormancy release, which were also involved in metabolic pathways.

**Additional file 4: Table S4.** Primer sequences used for qRT-PCR analysis in current study.

**Additional file 5: Table S5.** The accession number of protein used in phylogenetic analysis.

**Additional file 6: Figure S1.** OPLS-DA score plot of each comparison group.

**Additional file 7: Figure S2.** Venn diagram of 4 comparative metabolites. (a) up-regulated metabolites. (b) down-regulated metabolites.

**Additional file 8: Figure S3.** The KEGG enrichment analysis for top 20 of differential metabolites. Metabolites with a *P* value of T test of < 0.05 and VIP ≥ 1 were identified as differential metabolites between each two treatments.

**Additional file 9: Figure S4.** Phylogenetic analysis of related-gene used for expression analysis in this study. The amino acid sequences of proteins were aligned with Clustal W, and phylogenetic trees were constructed in MEGA 7 using Neighbor-Joining method with the following options: partial deletion and replicate bootstrap (1000). The protein accession number were shown in Table S5. (a) Sucrose-related genes. (b) Flavonoid-related genes. (c) Phytohormone-related genes.

## Data Availability

The datasets used and/or analysed during the current study available from the corresponding author on reasonable request. The sequences of the genes used in this study are available at Genbank (https://www.ncbi.nlm.nih.gov/genbank/), and the accession numbers are as follows: *PsCHS*, JN105300.1; *PsCHI*, ADK55061.1; *PsF3H*, HQ283447.1; *PsDFR*, HQ283448.1; *PsANS*, KJ466969.1; *Ps3GT*, MT702582; *PsAFB2*, MT702583; *PsTIR1*, MT702589; *PsMYC2*, MT702586; *PsCIN*, MT702584; *PsCWIN*, MT702585; *PsVIN*, MT702590; *PsSUS1*, MT702587; *PsSUS2*, MT702588.

## Abbreviations

QC: Quality control
OPLS-DA: Orthogonal projection to latent structures-discriminant analysis
DMs: Differential metabolites
SD: Standard deviation
VIP: Variable importance in the project
GC-MS/MS: Gas chromatography-mass spectrometry
LC-MS/MS: Liquid chromatography electrospray ionisation tandem mass spectrometry
EBG: Early biosynthetic gene of flavonoid biosynthesis
LBG: Late biosynthetic gene of flavonoid biosynthesis
bHLH: Basic helix-loop-helix protein
WD40: WD repeat protein
ANOVA: Analysis of variance
