# Supplementary figures and images for: Metabolomics analysis reveals Embden Meyerhof Parnas pathway activation and flavonoids accumulation during dormancy transition in tree peony
Source: BMC Plant Biol. 2020 Oct 23;20:484. doi: 10.1186/s12870-020-02692-x (PMC7583197; doi:10.1186/s12870-020-02692-x)

7d vs 0d

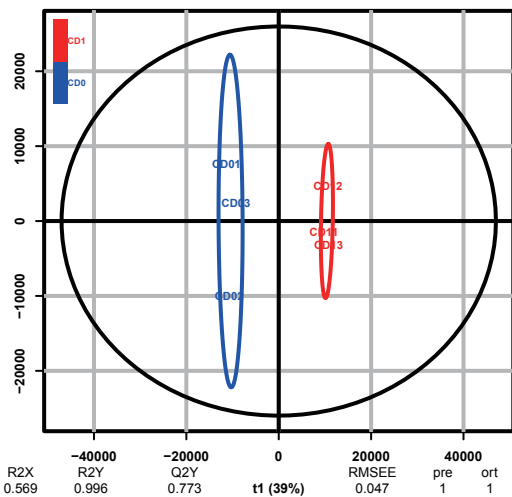

14d vs 0d

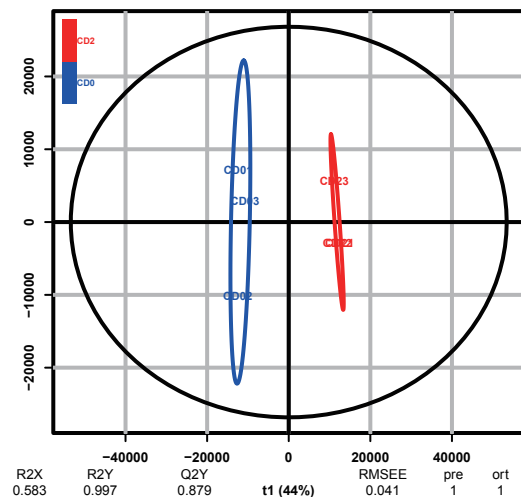

21d vs 0d

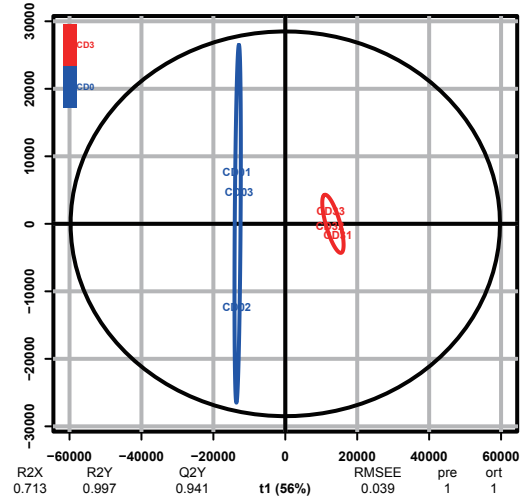

28d vs 0d

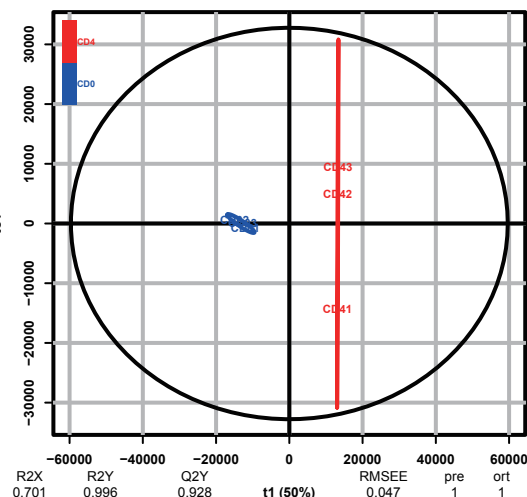

14d vs 7d

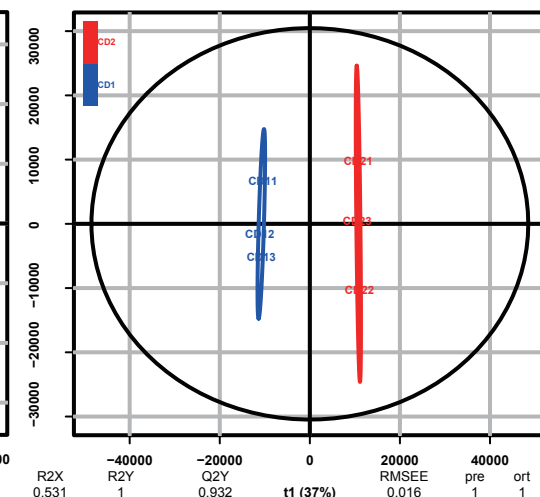

21d vs 7d

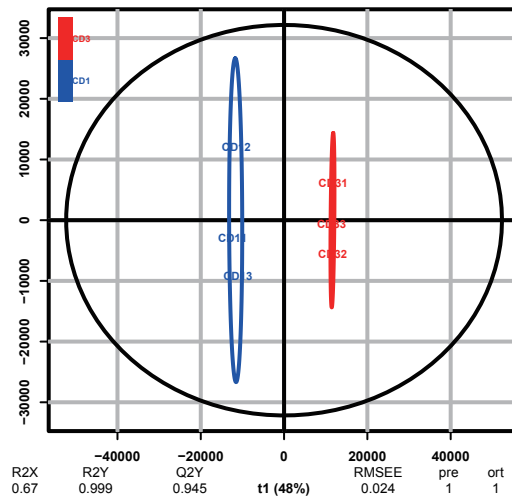

28d vs 7d

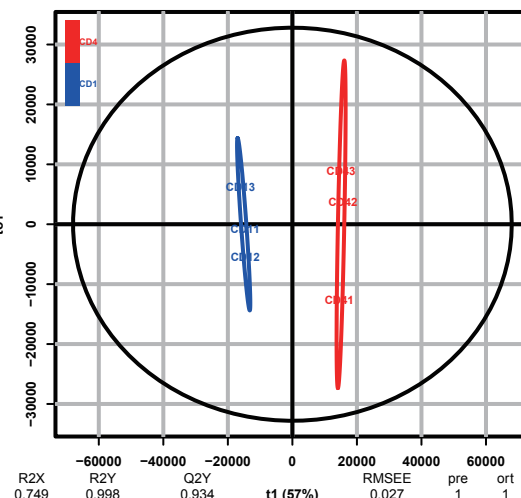

21d vs 14d

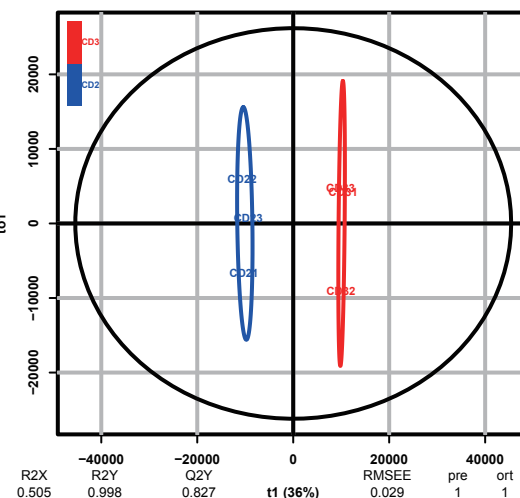

28d vs 14d

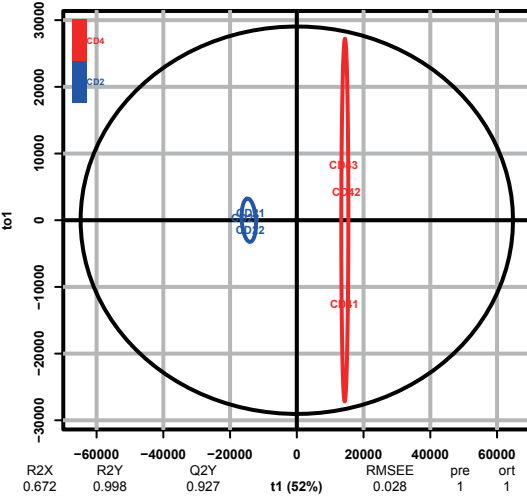

28d vs 21d

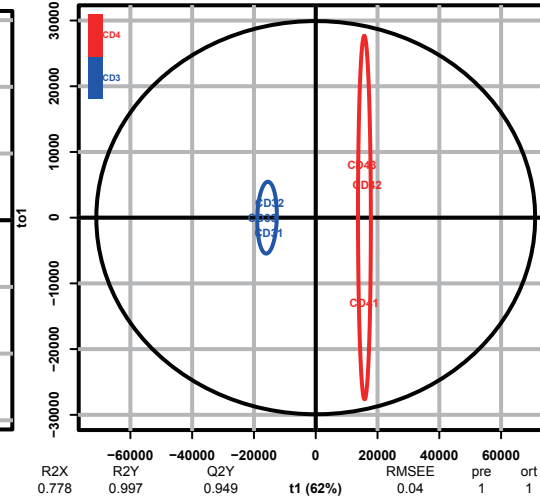

Supplement: Supplementary file 6 — Additional file 6: Figure S1. OPLS-DA score plot of each comparison group. [file 12870_2020_2692_MOESM6_ESM.pdf]

a

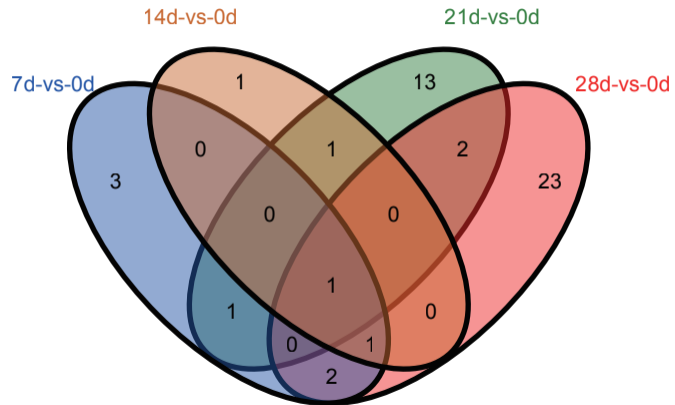

b

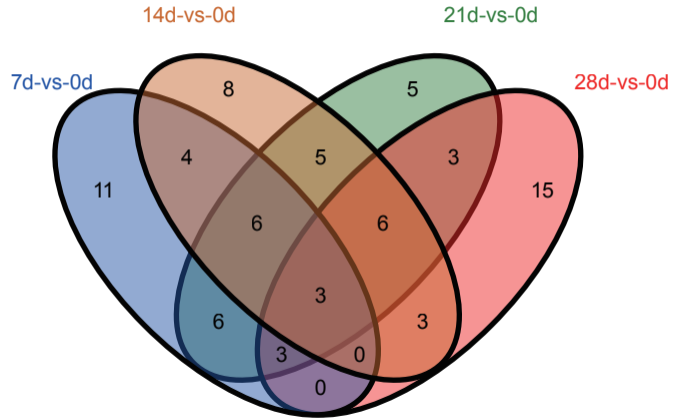

Supplement: Supplementary file 7 — Additional file 7: Figure S2. Venn diagram of 4 comparative metabolites. (a) up-regulated metabolites. (b) down-regulated metabolites. [file 12870_2020_2692_MOESM7_ESM.pdf]

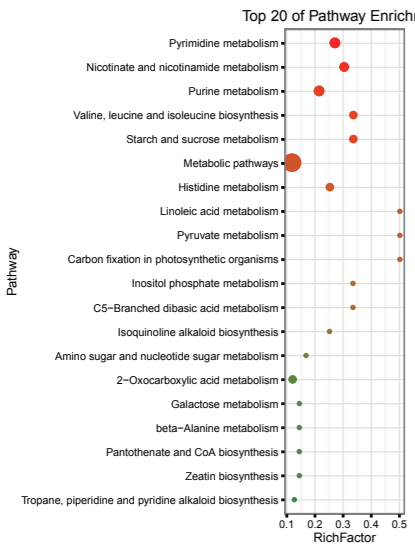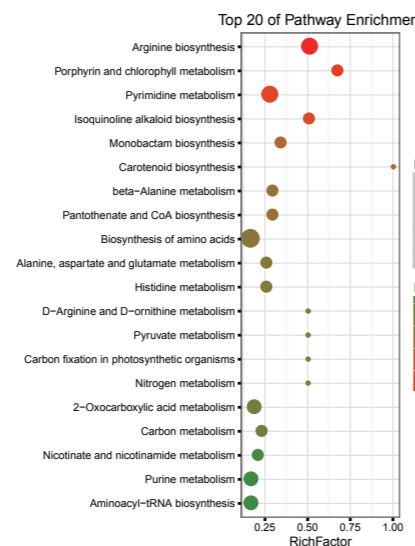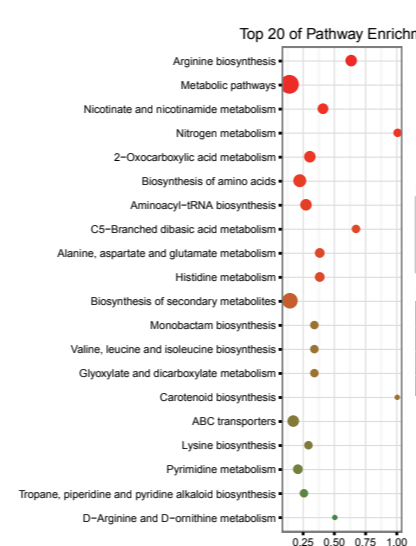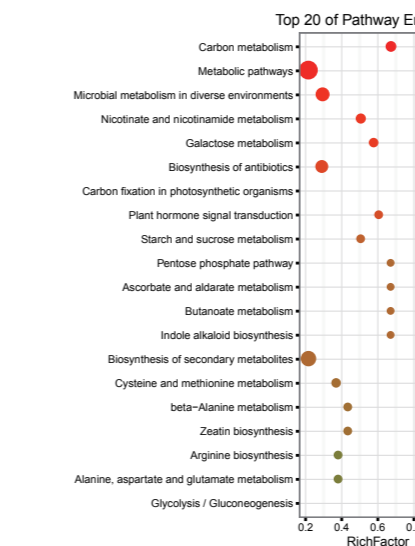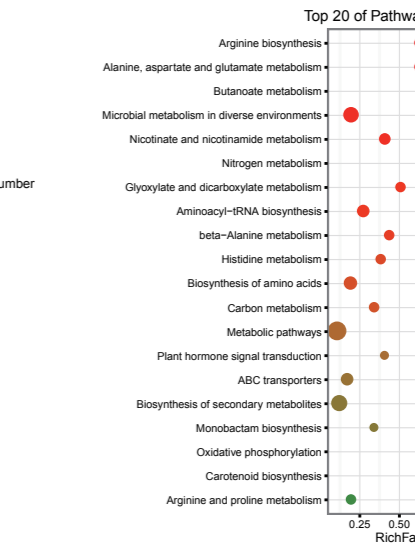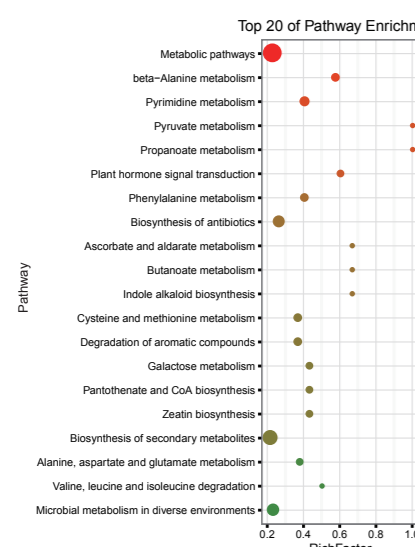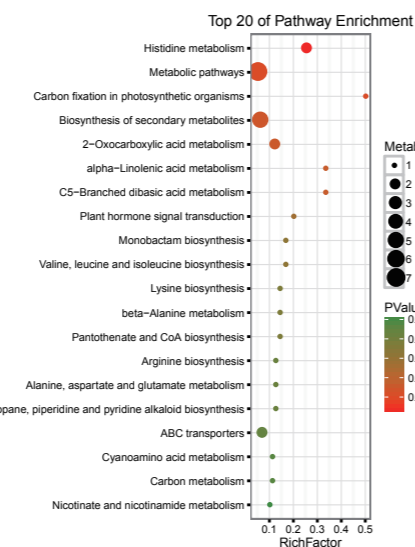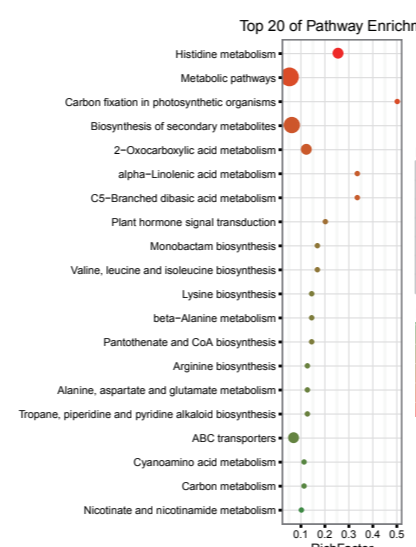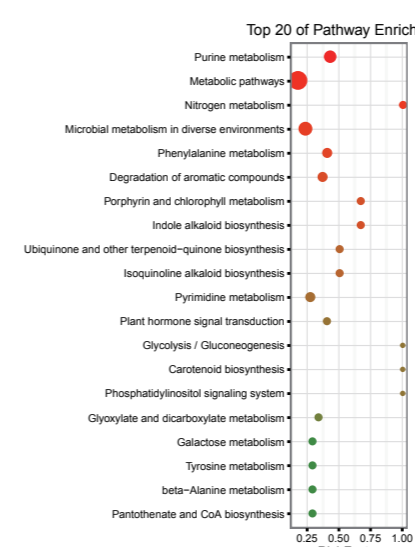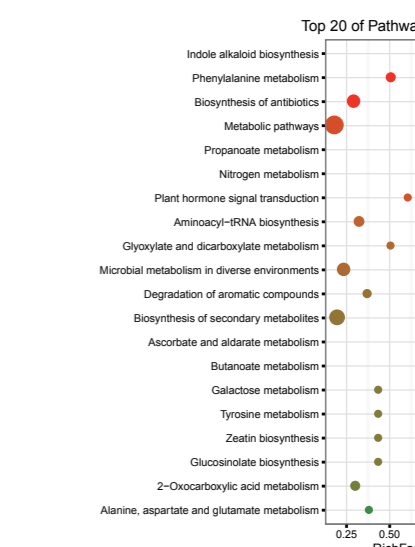

Supplement: Supplementary file 8 — Additional file 8: Figure S3. The KEGG enrichment analysis for top 20 of differential metabolites. Metabolites with a P value of T test of < 0.05 and VIP ≥ 1 were identified as differential metabolites between each two treatments. [file 12870_2020_2692_MOESM8_ESM.pdf]

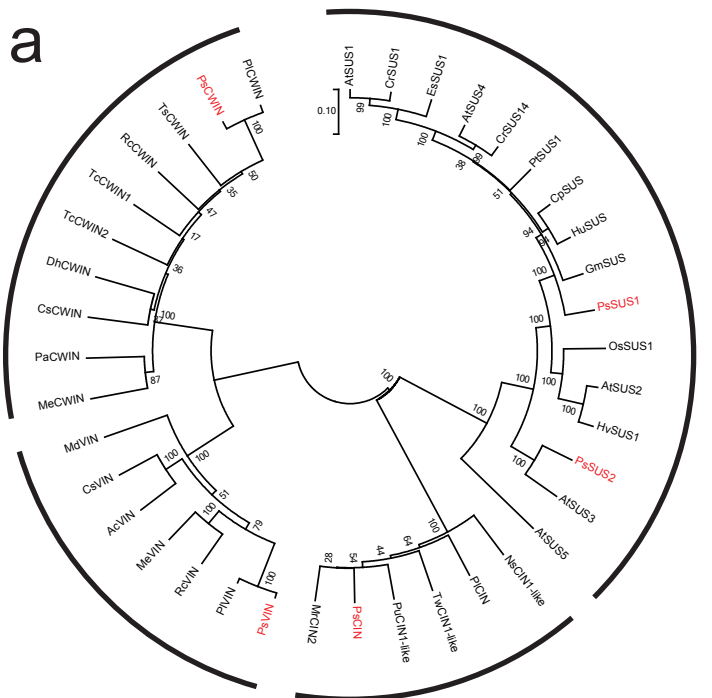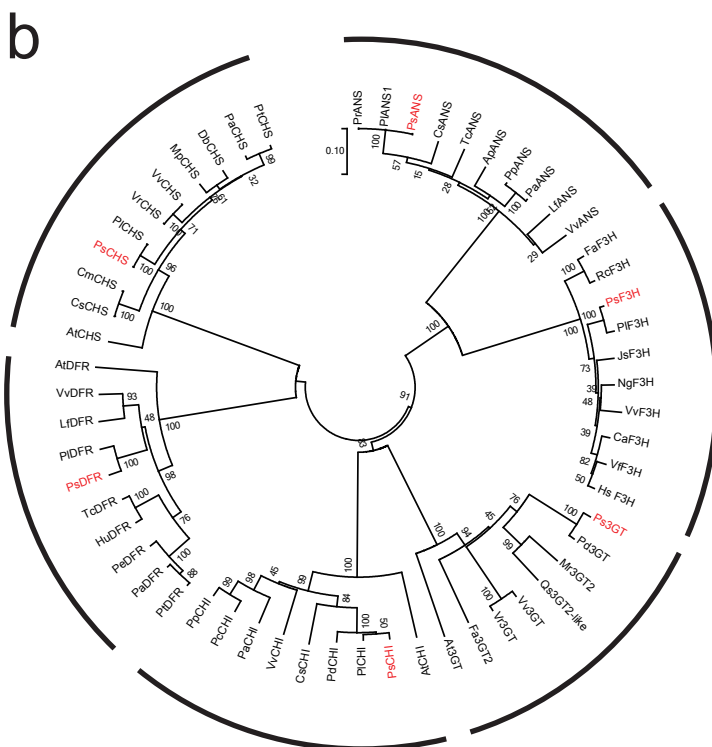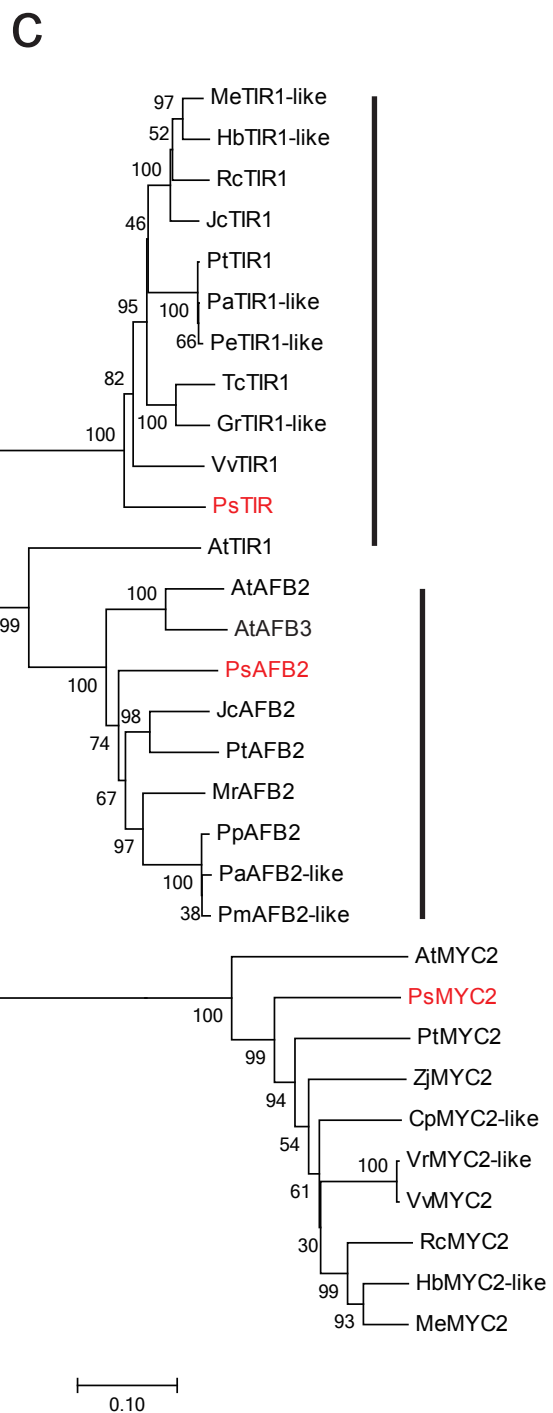

Supplement: Supplementary file 9 — Additional file 9: Figure S4. Phylogenetic analysis of related-gene used for expression analysis in this study. The amino acid sequences of proteins were aligned with Clustal W, and phylogenetic trees were constructed in MEGA 7 using Neighbor-Joining method with the following options: partial deletion and replicate bootstrap (1000). The protein accession number were shown in Table S5. (a) Sucrose-related genes. (b) Flavonoid-related genes. (c) Phytohormone-related genes. [file 12870_2020_2692_MOESM9_ESM.pdf]
